# 1,3-Bis(2-anilino-2-oxoeth­yl)-1*H*-imidazol-3-ium chloride dimethyl­formamide monosolvate

**DOI:** 10.1107/S1600536812045059

**Published:** 2012-11-03

**Authors:** Hon Man Lee, Jing-Yao Zeng

**Affiliations:** a Department of Chemistry, National Changhua University of Education, Changhua, Taiwan 50058

## Abstract

In the imidazolium cation of the title compound, C_19_H_19_N_4_O_2_^+^·Cl^−^·C_3_H_7_NO, the dihedral angles between the imidazole ring and the phenyl rings are 85.86 (4) and 70.26 (5)°. In the crystal, N—H⋯Cl hydrogen bonds link the imdiazo­lium cations and chloride anions into zigzag chains along [110] and together with C—H⋯Cl and C—H⋯O hydrogen bonds, which involve also the dimethyl­formamide solvent mol­ecule, form a two-dimensional network extending across the *ab* plane.

## Related literature

For the crystal structures of the non-solvated title compound and an acetonitrile monosolvate, see: Liao & Lee (2012 ▶) and Liao & Lee (2011 ▶), respectively. For the crystal structures of nickel, palladium, and silver complexes with ligands derived from the title compound, see: Liao, Chan, Chang *et al.* (2007 ▶), Liao, Chan, Zeng *et al.* (2007 ▶) and Liao *et al.* (2008 ▶), respectively.
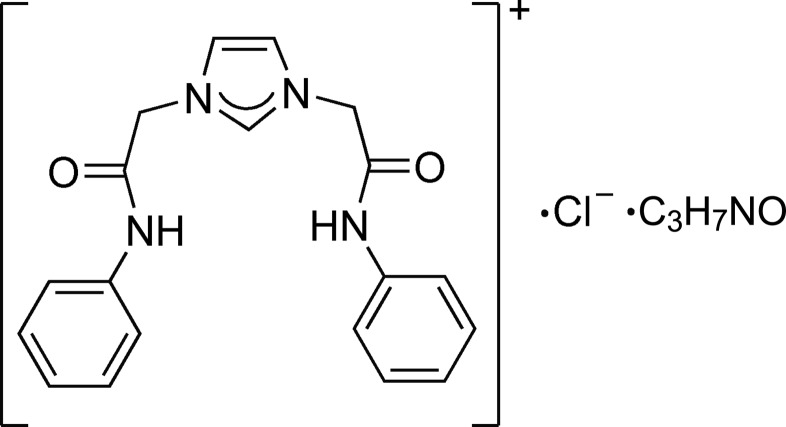


## Experimental

### 

#### Crystal data

C_19_H_19_N_4_O_2_^+^·Cl^−^·C_3_H_7_NO
*M* *_r_* = 443.93
Triclinic, 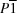

*a* = 9.2352 (5) Å
*b* = 9.9907 (5) Å
*c* = 14.0805 (7) Å
α = 109.119 (3)°
β = 96.342 (3)°
γ = 107.224 (3)°
*V* = 1141.05 (11) Å^3^
*Z* = 2
Mo *K*α radiation
μ = 0.20 mm^−1^
*T* = 150 K
0.50 × 0.32 × 0.22 mm

#### Data collection

Bruker SMART APEXII CCD diffractometer
Absorption correction: multi-scan (*SADABS*; Sheldrick, 2003 ▶) *T*_min_ = 0.883, *T*_max_ = 0.957
13404 measured reflections
5676 independent reflections
4849 reflections with *I* > 2σ(*I*)
*R*_int_ = 0.018

#### Refinement

*R*[*F* ^2^ > 2σ(*F* ^2^)] = 0.033
*wR*(*F* ^2^) = 0.091
*S* = 1.06
5676 reflections
282 parameters
H-atom parameters constrained
Δρ_max_ = 0.27 e Å^−3^
Δρ_min_ = −0.20 e Å^−3^

### 

Data collection: *APEX2* (Bruker, 2007 ▶); cell refinement: *SAINT* (Bruker, 2007 ▶); data reduction: *SAINT*; program(s) used to solve structure: *SHELXTL* (Sheldrick, 2008 ▶); program(s) used to refine structure: *SHELXTL*; molecular graphics: *SHELXTL*; software used to prepare material for publication: *DIAMOND* (Brandenburg, 2006 ▶).

## Supplementary Material

Crystal structure: contains datablock(s) I, global. DOI: 10.1107/S1600536812045059/zs2241sup1.cif

Structure factors: contains datablock(s) I. DOI: 10.1107/S1600536812045059/zs2241Isup2.hkl

Supplementary material file. DOI: 10.1107/S1600536812045059/zs2241Isup3.cml

Additional supplementary materials:  crystallographic information; 3D view; checkCIF report

## Figures and Tables

**Table 1:** Hydrogen-bond geometry (Å, °)

*D*—H⋯*A*	*D*—H	H⋯*A*	*D*⋯*A*	*D*—H⋯*A*
N4—H4⋯Cl1^i^	0.88	2.39	3.2696 (10)	174
N5—H5⋯Cl1^ii^	0.88	2.35	3.2292 (10)	172
C1—H1⋯O4^iii^	0.95	2.32	3.0910 (14)	138
C2—H2⋯O3^iv^	0.95	2.49	3.1619 (13)	128
C12—H12*A*⋯Cl1	0.99	2.63	3.4269 (12)	137
C12—H12*B*⋯O3^iv^	0.99	2.44	3.1544 (14)	129
C20—H20*A*⋯O4^iv^	0.99	2.52	3.2790 (15)	133
C23—H23*A*⋯O1^iv^	0.98	2.53	3.3472 (18)	141

Symmetry codes: (i) 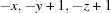; (ii) 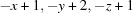; (iii) 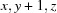; (iv) 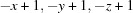.

**Fig. 1.**
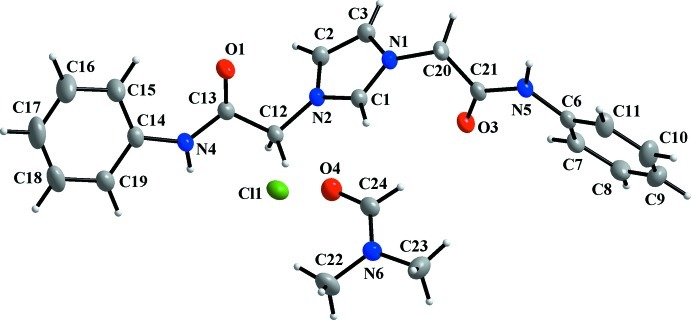
The structure of the title compound, showing 50% probability displacement ellipsoids for the non-hydrogen atoms. The H atoms are shown as spheres of arbitrary radius.

**Fig. 2.**
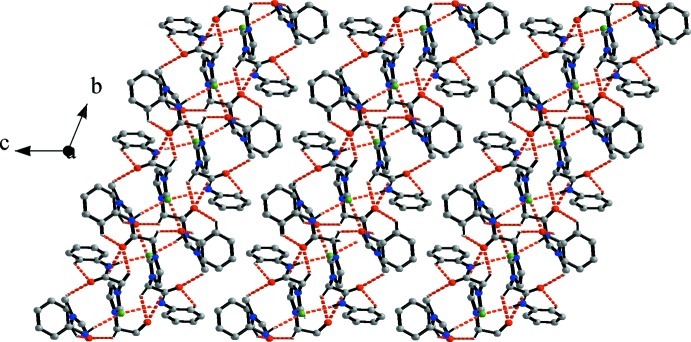
The crystal packing viewed down the a axis, displaying the hydrogen bonds as dashed lines.

## supplementary crystallographic information

### Comment

The title compound, C_19_H_19_N_4_O_2_^+^.Cl^-^.C_3_H_7_NO, is the dimethylformamide solvate of a reliable ligand precusor for the preparation of transition metal complexes with *N*-heterocyclic carbene (NHC) ligands. We reported previously the crystal structures of the non-solvated title compound (Liao & Lee 2012) and an acetonitrile monosolvate (Liao & Lee 2011). Transition metal complexes with NHC ligands obtained from the title compound include those with nickel(II) (Liao, Chan, Chang *et al.* 2007), palladium(II) (Liao, Chan, Zeng *et al.* 2007) and silver(I) (Liao *et al.* 2008).

In the structure of the title compound (Fig. 1), the dihedral angles between the heterocyclic ring and the two phenyl rings in the imdiaozlium cation.are 85.86 (4)° and 70.26 (5)° and the molecular conformation is stabilized by intramolecular C7—H···O3 and C15—H···O1 interations. In the crystal, classical intermolecular hydrogen bonds of the type N—H···Cl (Table 1) involving both N4 and N5 link the cations into zigzag chains along the [110] direction and together with non-classical C—H···O and C—H···Cl hydrogen bonds further connect these chains and the DMF solvent molecules into two-dimensional layers lying on the *ab* plane (Fig. 2).

### Experimental

The compound was prepared according to the literature procedure (Liao, Chan, Zeng *et al.*, 2007). Suitable crystals were obtained by slow diffusion of diethyl ether into a DMF solution of the compound at room temperature.

### Refinement

All of the hydrogen atoms could have been discerned in the difference-Fourier map but were positioned geometrically and refined as riding atoms, with C_aryl_—H = 0.95, C_methyl_—H = 0.98, C_methylene_—H = 0.99, C_methine_—H = 0.95, and NH = 0.88 Å , with *U*_iso_(H) = 1.2*U*_eq_ (C_aryl_, C_methylene_, C_methine_ and N) and *U*_iso_(H) = 1.5 *U*_eq_ (C_methyl_).

### Crystal data

**Table:**

C_19_H_19_N_4_O_2_^+^·Cl^−^·C_3_H_7_NO	*Z* = 2
*M**_r_* = 443.93	*F*(000) = 468
Triclinic, *P*1	*D*_x_ = 1.292 Mg m^−^^3^
Hall symbol: -P 1	Mo *K*α radiation, λ = 0.71073 Å
*a* = 9.2352 (5) Å	Cell parameters from 6152 reflections
*b* = 9.9907 (5) Å	θ = 2.3–28.3°
*c* = 14.0805 (7) Å	µ = 0.20 mm^−^^1^
α = 109.119 (3)°	*T* = 150 K
β = 96.342 (3)°	Plate, colourless
γ = 107.224 (3)°	0.50 × 0.32 × 0.22 mm
*V* = 1141.05 (11) Å^3^	

### Data collection

**Table:**

Bruker SMART APEXII CCD diffractometer	5676 independent reflections
Radiation source: fine-focus sealed tube	4849 reflections with *I* > 2σ(*I*)
Graphite monochromator	*R*_int_ = 0.018
ω scans	θ_max_ = 28.3°, θ_min_ = 2.3°
Absorption correction: multi-scan (*SADABS*; Sheldrick, 2003)	*h* = −12→12
*T*_min_ = 0.883, *T*_max_ = 0.957	*k* = −13→13
13404 measured reflections	*l* = −18→17

### Refinement

**Table:**

Refinement on *F*^2^	Primary atom site location: structure-invariant direct methods
Least-squares matrix: full	Secondary atom site location: difference Fourier map
*R*[*F*^2^ > 2σ(*F*^2^)] = 0.033	Hydrogen site location: inferred from neighbouring sites
*wR*(*F*^2^) = 0.091	H-atom parameters constrained
*S* = 1.06	*w* = 1/[σ^2^(*F*_o_^2^) + (0.0418*P*)^2^ + 0.3283*P*] where *P* = (*F*_o_^2^ + 2*F*_c_^2^)/3
5676 reflections	(Δ/σ)_max_ = 0.002
282 parameters	Δρ_max_ = 0.27 e Å^−^^3^
0 restraints	Δρ_min_ = −0.20 e Å^−^^3^

### Special details

**Table:**

Geometry. All e.s.d.'s (except the e.s.d. in the dihedral angle between two l.s. planes) are estimated using the full covariance matrix. The cell e.s.d.'s are taken into account individually in the estimation of e.s.d.'s in distances, angles and torsion angles; correlations between e.s.d.'s in cell parameters are only used when they are defined by crystal symmetry. An approximate (isotropic) treatment of cell e.s.d.'s is used for estimating e.s.d.'s involving l.s. planes.
Refinement. Refinement of *F*^2^ against ALL reflections. The weighted *R*-factor *wR* and goodness of fit *S* are based on *F*^2^, conventional *R*-factors *R* are based on *F*, with *F* set to zero for negative *F*^2^. The threshold expression of *F*^2^ > σ(*F*^2^) is used only for calculating *R*-factors(gt) *etc*. and is not relevant to the choice of reflections for refinement. *R*-factors based on *F*^2^ are statistically about twice as large as those based on *F*, and *R*- factors based on ALL data will be even larger.

### Fractional atomic coordinates and isotropic or equivalent isotropic displacement parameters (Å^2^)

**Table:**

	*x*	*y*	*z*	*U*_iso_*/*U*_eq_	
C1	0.45911 (12)	0.71326 (12)	0.46996 (8)	0.0221 (2)	
H1	0.4207	0.7855	0.5108	0.027*	
C2	0.47374 (13)	0.49834 (12)	0.36866 (9)	0.0242 (2)	
H2	0.4457	0.3943	0.3265	0.029*	
C3	0.61439 (13)	0.60839 (12)	0.39059 (9)	0.0242 (2)	
H3	0.7040	0.5962	0.3672	0.029*	
C6	0.96547 (12)	1.12971 (12)	0.77366 (9)	0.0242 (2)	
C7	0.99535 (14)	1.04413 (14)	0.82886 (10)	0.0310 (3)	
H7	0.9590	0.9368	0.7969	0.037*	
C8	1.07892 (16)	1.11709 (17)	0.93125 (11)	0.0392 (3)	
H8	1.1012	1.0590	0.9686	0.047*	
C9	1.12987 (17)	1.27310 (18)	0.97937 (11)	0.0438 (3)	
H9	1.1858	1.3219	1.0495	0.053*	
C10	1.09870 (18)	1.35748 (16)	0.92440 (12)	0.0443 (3)	
H10	1.1332	1.4646	0.9573	0.053*	
C11	1.01746 (15)	1.28717 (14)	0.82159 (11)	0.0334 (3)	
H11	0.9975	1.3460	0.7842	0.040*	
C12	0.21858 (12)	0.48847 (12)	0.42117 (9)	0.0228 (2)	
H12A	0.1854	0.5583	0.4746	0.027*	
H12B	0.2138	0.4004	0.4401	0.027*	
C13	0.10731 (13)	0.43442 (12)	0.31724 (9)	0.0229 (2)	
C14	−0.17305 (13)	0.28559 (12)	0.23667 (9)	0.0265 (2)	
C15	−0.17370 (16)	0.27958 (17)	0.13651 (11)	0.0406 (3)	
H15	−0.0785	0.3132	0.1169	0.049*	
C16	−0.31538 (19)	0.2237 (2)	0.06535 (13)	0.0543 (4)	
H16	−0.3164	0.2187	−0.0033	0.065*	
C17	−0.45506 (17)	0.17540 (18)	0.09346 (14)	0.0509 (4)	
H17	−0.5511	0.1409	0.0450	0.061*	
C18	−0.45412 (16)	0.17760 (16)	0.19224 (13)	0.0438 (3)	
H18	−0.5495	0.1416	0.2111	0.053*	
C19	−0.31349 (15)	0.23256 (15)	0.26377 (11)	0.0350 (3)	
H19	−0.3130	0.2340	0.3316	0.042*	
C20	0.72883 (13)	0.88905 (12)	0.50232 (9)	0.0242 (2)	
H20A	0.6916	0.9693	0.4951	0.029*	
H20B	0.8162	0.8883	0.4675	0.029*	
C21	0.78517 (12)	0.92260 (12)	0.61644 (9)	0.0224 (2)	
C22	0.33318 (17)	0.13837 (18)	0.76265 (14)	0.0485 (4)	
H22A	0.2929	0.0459	0.7000	0.073*	
H22B	0.2750	0.2049	0.7590	0.073*	
H22C	0.3210	0.1118	0.8232	0.073*	
C23	0.57161 (19)	0.36610 (16)	0.85242 (11)	0.0437 (3)	
H23A	0.6817	0.4056	0.8512	0.066*	
H23B	0.5633	0.3596	0.9197	0.066*	
H23C	0.5201	0.4341	0.8410	0.066*	
C24	0.57427 (14)	0.15345 (14)	0.70631 (10)	0.0287 (2)	
H24	0.6815	0.2093	0.7163	0.034*	
Cl1	0.04385 (3)	0.75405 (3)	0.48455 (2)	0.02976 (8)	
N1	0.60237 (11)	0.74204 (10)	0.45370 (7)	0.02214 (19)	
N2	0.37871 (10)	0.56600 (10)	0.41932 (7)	0.02100 (18)	
N4	−0.03447 (11)	0.33950 (11)	0.31377 (7)	0.0248 (2)	
H4	−0.0412	0.3078	0.3651	0.030*	
N5	0.89023 (11)	1.06440 (10)	0.66757 (7)	0.02343 (19)	
H5	0.9141	1.1215	0.6314	0.028*	
N6	0.49695 (12)	0.21613 (12)	0.77134 (8)	0.0311 (2)	
O1	0.14435 (10)	0.47707 (10)	0.24851 (7)	0.0329 (2)	
O3	0.73894 (10)	0.82875 (9)	0.65467 (7)	0.02789 (18)	
O4	0.51865 (11)	0.02961 (10)	0.63458 (7)	0.0347 (2)	

### Atomic displacement parameters (Å^2^)

**Table:**

	*U*^11^	*U*^22^	*U*^33^	*U*^12^	*U*^13^	*U*^23^
C1	0.0213 (5)	0.0193 (5)	0.0242 (5)	0.0060 (4)	0.0035 (4)	0.0081 (4)
C2	0.0243 (5)	0.0198 (5)	0.0264 (5)	0.0085 (4)	0.0042 (4)	0.0062 (4)
C3	0.0232 (5)	0.0231 (5)	0.0267 (5)	0.0097 (4)	0.0048 (4)	0.0087 (4)
C6	0.0173 (5)	0.0235 (5)	0.0273 (5)	0.0039 (4)	0.0055 (4)	0.0070 (4)
C7	0.0268 (6)	0.0288 (6)	0.0323 (6)	0.0009 (5)	0.0044 (5)	0.0139 (5)
C8	0.0329 (7)	0.0482 (8)	0.0321 (7)	0.0020 (6)	0.0057 (5)	0.0214 (6)
C9	0.0382 (7)	0.0515 (8)	0.0258 (6)	0.0051 (6)	0.0045 (6)	0.0054 (6)
C10	0.0399 (8)	0.0323 (7)	0.0411 (8)	0.0093 (6)	0.0010 (6)	−0.0047 (6)
C11	0.0283 (6)	0.0246 (6)	0.0396 (7)	0.0089 (5)	0.0014 (5)	0.0050 (5)
C12	0.0187 (5)	0.0229 (5)	0.0255 (5)	0.0043 (4)	0.0038 (4)	0.0104 (4)
C13	0.0212 (5)	0.0213 (5)	0.0253 (5)	0.0067 (4)	0.0043 (4)	0.0088 (4)
C14	0.0214 (5)	0.0226 (5)	0.0304 (6)	0.0050 (4)	0.0018 (4)	0.0073 (4)
C15	0.0293 (7)	0.0486 (8)	0.0348 (7)	0.0011 (6)	0.0003 (5)	0.0180 (6)
C16	0.0431 (9)	0.0620 (10)	0.0418 (8)	0.0000 (7)	−0.0096 (7)	0.0227 (8)
C17	0.0296 (7)	0.0474 (8)	0.0568 (10)	0.0047 (6)	−0.0129 (7)	0.0117 (7)
C18	0.0218 (6)	0.0399 (7)	0.0531 (9)	0.0055 (5)	0.0044 (6)	0.0034 (6)
C19	0.0253 (6)	0.0346 (6)	0.0349 (7)	0.0051 (5)	0.0076 (5)	0.0051 (5)
C20	0.0208 (5)	0.0186 (5)	0.0282 (5)	0.0016 (4)	0.0032 (4)	0.0083 (4)
C21	0.0173 (5)	0.0197 (5)	0.0289 (5)	0.0056 (4)	0.0048 (4)	0.0086 (4)
C22	0.0321 (7)	0.0494 (8)	0.0585 (10)	0.0109 (6)	0.0224 (7)	0.0128 (7)
C23	0.0502 (9)	0.0397 (7)	0.0298 (7)	0.0094 (6)	0.0076 (6)	0.0054 (6)
C24	0.0228 (5)	0.0331 (6)	0.0324 (6)	0.0101 (5)	0.0065 (5)	0.0147 (5)
Cl1	0.03040 (15)	0.02890 (14)	0.03696 (16)	0.01106 (11)	0.01239 (12)	0.01916 (12)
N1	0.0201 (4)	0.0190 (4)	0.0248 (4)	0.0049 (3)	0.0028 (4)	0.0078 (4)
N2	0.0188 (4)	0.0193 (4)	0.0235 (4)	0.0056 (3)	0.0025 (3)	0.0082 (3)
N4	0.0207 (5)	0.0260 (4)	0.0253 (5)	0.0036 (4)	0.0030 (4)	0.0117 (4)
N5	0.0212 (4)	0.0192 (4)	0.0275 (5)	0.0028 (3)	0.0034 (4)	0.0103 (4)
N6	0.0268 (5)	0.0322 (5)	0.0307 (5)	0.0083 (4)	0.0082 (4)	0.0090 (4)
O1	0.0255 (4)	0.0406 (5)	0.0300 (4)	0.0028 (4)	0.0039 (3)	0.0189 (4)
O3	0.0264 (4)	0.0218 (4)	0.0321 (4)	0.0024 (3)	0.0044 (3)	0.0122 (3)
O4	0.0314 (5)	0.0316 (4)	0.0381 (5)	0.0131 (4)	0.0075 (4)	0.0078 (4)

### Geometric parameters (Å, º)

**Table:**

C1—N1	1.3306 (14)	C15—C16	1.392 (2)
C1—N2	1.3316 (13)	C15—H15	0.9500
C1—H1	0.9500	C16—C17	1.387 (2)
C2—C3	1.3545 (16)	C16—H16	0.9500
C2—N2	1.3819 (14)	C17—C18	1.383 (2)
C2—H2	0.9500	C17—H17	0.9500
C3—N1	1.3846 (14)	C18—C19	1.3889 (19)
C3—H3	0.9500	C18—H18	0.9500
C6—C7	1.3924 (17)	C19—H19	0.9500
C6—C11	1.3951 (16)	C20—N1	1.4624 (13)
C6—N5	1.4155 (15)	C20—C21	1.5245 (16)
C7—C8	1.3922 (18)	C20—H20A	0.9900
C7—H7	0.9500	C20—H20B	0.9900
C8—C9	1.383 (2)	C21—O3	1.2229 (13)
C8—H8	0.9500	C21—N5	1.3529 (14)
C9—C10	1.384 (2)	C22—N6	1.4515 (17)
C9—H9	0.9500	C22—H22A	0.9800
C10—C11	1.391 (2)	C22—H22B	0.9800
C10—H10	0.9500	C22—H22C	0.9800
C11—H11	0.9500	C23—N6	1.4552 (17)
C12—N2	1.4598 (14)	C23—H23A	0.9800
C12—C13	1.5201 (15)	C23—H23B	0.9800
C12—H12A	0.9900	C23—H23C	0.9800
C12—H12B	0.9900	C24—O4	1.2268 (15)
C13—O1	1.2215 (14)	C24—N6	1.3319 (16)
C13—N4	1.3550 (14)	C24—H24	0.9500
C14—C15	1.3912 (18)	N4—H4	0.8800
C14—C19	1.3939 (17)	N5—H5	0.8800
C14—N4	1.4172 (14)		
			
N1—C1—N2	108.43 (9)	C16—C17—H17	120.1
N1—C1—H1	125.8	C17—C18—C19	119.86 (14)
N2—C1—H1	125.8	C17—C18—H18	120.1
C3—C2—N2	107.04 (9)	C19—C18—H18	120.1
C3—C2—H2	126.5	C18—C19—C14	120.44 (13)
N2—C2—H2	126.5	C18—C19—H19	119.8
C2—C3—N1	106.80 (10)	C14—C19—H19	119.8
C2—C3—H3	126.6	N1—C20—C21	110.01 (9)
N1—C3—H3	126.6	N1—C20—H20A	109.7
C7—C6—C11	119.90 (11)	C21—C20—H20A	109.7
C7—C6—N5	122.69 (10)	N1—C20—H20B	109.7
C11—C6—N5	117.33 (11)	C21—C20—H20B	109.7
C8—C7—C6	119.49 (12)	H20A—C20—H20B	108.2
C8—C7—H7	120.3	O3—C21—N5	125.42 (11)
C6—C7—H7	120.3	O3—C21—C20	122.24 (10)
C9—C8—C7	120.86 (13)	N5—C21—C20	112.34 (9)
C9—C8—H8	119.6	N6—C22—H22A	109.5
C7—C8—H8	119.6	N6—C22—H22B	109.5
C8—C9—C10	119.40 (13)	H22A—C22—H22B	109.5
C8—C9—H9	120.3	N6—C22—H22C	109.5
C10—C9—H9	120.3	H22A—C22—H22C	109.5
C9—C10—C11	120.73 (13)	H22B—C22—H22C	109.5
C9—C10—H10	119.6	N6—C23—H23A	109.5
C11—C10—H10	119.6	N6—C23—H23B	109.5
C10—C11—C6	119.62 (13)	H23A—C23—H23B	109.5
C10—C11—H11	120.2	N6—C23—H23C	109.5
C6—C11—H11	120.2	H23A—C23—H23C	109.5
N2—C12—C13	111.82 (9)	H23B—C23—H23C	109.5
N2—C12—H12A	109.3	O4—C24—N6	125.49 (12)
C13—C12—H12A	109.3	O4—C24—H24	117.3
N2—C12—H12B	109.3	N6—C24—H24	117.3
C13—C12—H12B	109.3	C1—N1—C3	108.89 (9)
H12A—C12—H12B	107.9	C1—N1—C20	124.81 (9)
O1—C13—N4	126.11 (11)	C3—N1—C20	126.11 (10)
O1—C13—C12	122.49 (10)	C1—N2—C2	108.84 (9)
N4—C13—C12	111.38 (9)	C1—N2—C12	125.25 (9)
C15—C14—C19	119.73 (12)	C2—N2—C12	125.86 (9)
C15—C14—N4	123.11 (11)	C13—N4—C14	127.22 (10)
C19—C14—N4	117.14 (11)	C13—N4—H4	116.4
C14—C15—C16	119.31 (14)	C14—N4—H4	116.4
C14—C15—H15	120.3	C21—N5—C6	126.27 (9)
C16—C15—H15	120.3	C21—N5—H5	116.9
C17—C16—C15	120.81 (15)	C6—N5—H5	116.9
C17—C16—H16	119.6	C24—N6—C22	121.12 (11)
C15—C16—H16	119.6	C24—N6—C23	121.65 (11)
C18—C17—C16	119.81 (14)	C22—N6—C23	117.22 (12)
C18—C17—H17	120.1		

### Hydrogen-bond geometry (Å, º)

**Table:**

*D*—H···*A*	*D*—H	H···*A*	*D*···*A*	*D*—H···*A*
N4—H4···Cl1^i^	0.88	2.39	3.2696 (10)	174
N5—H5···Cl1^ii^	0.88	2.35	3.2292 (10)	172
C1—H1···O4^iii^	0.95	2.32	3.0910 (14)	138
C2—H2···O3^iv^	0.95	2.49	3.1619 (13)	128
C12—H12*A*···Cl1	0.99	2.63	3.4269 (12)	137
C12—H12*B*···O3^iv^	0.99	2.44	3.1544 (14)	129
C20—H20*A*···O4^iv^	0.99	2.52	3.2790 (15)	133
C23—H23*A*···O1^iv^	0.98	2.53	3.3472 (18)	141
C22—H22*A*···O4	0.98	2.40	2.8049 (18)	104
C15—H15···O1	0.95	2.35	2.9178 (16)	118
C7—H7···O3	0.95	2.39	2.9147 (15)	115

Symmetry codes: (i) −*x*, −*y*+1, −*z*+1; (ii) −*x*+1, −*y*+2, −*z*+1; (iii) *x*, *y*+1, *z*; (iv) −*x*+1, −*y*+1, −*z*+1.
